# Substrate dependent *in vitro* antifungal activity of *Bacillus sp* strain AR2

**DOI:** 10.1186/1475-2859-13-67

**Published:** 2014-05-14

**Authors:** Anil Kumar Singh, Ria Rautela, Swaranjit Singh Cameotra

**Affiliations:** 1 Institute of Microbial Technology, Sector 39 A, Chandigarh 160036, India

**Keywords:** Biosurfactant, Lipopeptides, Filamentous fungi, Fungicidal

## Abstract

**Background:**

Biosurfactants are a structurally diverse group of secondary metabolites with lots of potential to serve mankind. Depending upon the structure and composition they may exhibit properties that make them suitable for a particular application. Structural and compositional diversity of biosurfactant is unambiguously substrate dependent. The present study investigates the qualitative and quantitative effect of different water soluble carbon source on the biosurfactant produced by *Bacillus amylofaciens* strain AR2.

**Results:**

Strain AR2 produced lipopeptide type biosurfactant while growing on water soluble carbon sources. Maximum biosurfactant production was observed in the sucrose supplemented minimal salt medium (MSM). Strain AR2 exhibited carbon source dependent surface tension reduction in the range of 30-37 mN/m, critical micelle concentration (CMC) in the range 80-110 mg/l and emulsification index (EI_24_ kerosene) in the range of 32-66%. In dextrose, sucrose and glycerol supplemented MSM, strain AR2 produced lipopeptides as a mixture of surfactin, iturin and fengycin. However, in the presence of maltose, lactose and sorbitol only iturin was produced. This substrate dependent compositional variation in the lipopeptides significantly influenced antifungal activity. Lipopeptides produced by strain AR2 while growing on sucrose and dextrose based MSM was observed to be most efficient as an antifungal agent.

**Conclusions:**

These results suggest that carbon source provided for the growth and biosurfactant production not only influences the yield but also the type of biosurfactant. Sucrose is the most suitable carbon source for production of lipopeptide biosurfactant with antifungal activity.

## Background

Microbial surfactants are environment friendly alternative to synthetic surfactants. Microorganisms produce surfactant as a mixture of homologues and isoforms. In the recent past, biosurfactants have received considerable attention owing to their diverse biological activities [1,2] and potential commercial applications [3,4]. Biosurfactants particularly cyclic lipopeptides have been appreciated for its potential to serve the biomedical and pharmaceutical applications as an antimicrobial, antiadhesive, antimycoplasm, immunosuppressive and antitumor agent [4,5].

Cyclic lipopeptides are the product of non-ribosomal peptide synthetases or hybrid of polyketide synthases and non- ribosomal peptide synthetases. These modules of synthesis convey considerable structural diversity in the cyclic lipopeptides. Differences may emerge in the length and composition of the lipid moiety as well as in the type, number and configuration of the amino acids present in the peptide chain [5]. Structural diversity of lipopeptides can influence its physiochemical properties and biological activities significantly [6-8].

Carbon source is an important factor influencing microbial growth and biosurfactant production [9]. Water soluble as well as water immiscible substrates has been reported for biosurfactant production [10]. For *Bacillus* species water soluble substrates has been reported to be better than water immiscible substrates [11]. Most of the studies reported in literature have highlighted the effect of carbon source on biosurfactant yield. There are very few reports exhibiting effect of carbon source on the type of lipopeptide biosurfactant produced by the microorganism [6,7]. Good amount of superior quality biosurfactant is indispensable for outcompeting synthetic surfactants.

The goal of the current research is to examine the influence of different water soluble carbon source on lipopeptides produced by *Bacillus amyloliquefaciens* strain AR2. Various properties like surface tension reducing ability, critical micelle concentration (CMC), emulsification index (EI_24_) and antifungal activity of lipopeptides were studied to scrutinize the effect of different carbon source on the biosurfactant. Further, detailed composition analysis of biosurfactant was performed to explain the carbon source dependent changes observed in the properties.

## Results

### Effect of different carbon source on growth and biosurfactant production

**Figure 1:**
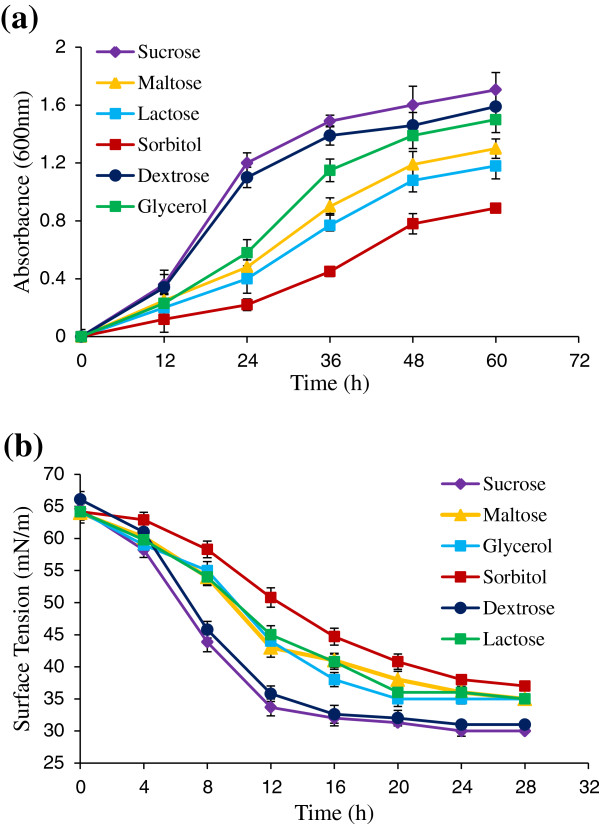
**Time course of (a) growth and (b) surface tension reduction of culture media by *B. amyloliquefaciens *strain AR2.** Values given are mean ± SD of five independent experiments.

The bacterium *B. amyloliquefaciens* strain AR2 exhibited different assimilation preference for different water soluble carbon sources provided for the growth. This was evident from the different growth pattern observed in the study. Carbon source namely sucrose and dextrose supported good growth as compared to other carbon sources namely glycerol, maltose, sorbitol and lactose. In dextrose and sucrose containing growth medium the log phase of strain AR2 ended with in 40 h of growth with very short lag phase. However, in the presence of other carbon source duration of lag phase increased and consequently, the duration of log phase got extended to 60 h (Figure 1a). After 64–68 h of growth strain AR2 entered the death phase as decline in growth curve was observed.

Growth of the bacterium was accompanied by decrease in the surface tension of growth media. Biosurfactant production as evident from decrease in the surface tension of culture media started within 6 h of inoculation (Figure 1b). Maximum decrease in the surface tension was observed after 24 h of growth. Depending upon the carbon source supplied for the growth, strain AR2 was able to reduce the surface tension of culture media in the range of 30-37 mN/m. The least surface tension value recorded among all the carbon source at the end of fermentation was 30 mN/m when sucrose was provided as a carbon substrate. Emulsification activity was observed in the range of 66-32% depending upon the carbon substrate. CMC value was also influenced by the carbon source provided for growth (Table 1). The most efficient biosurfactant was produced by strain AR2 while growing on sucrose and dextrose supplemented medium as CMC value of 80 mg/l was observed. Even though the strain AR2 was supplied with equal number of carbon in growth media, cell density and biosurfactant concentration was different for different carbon sources (Table 1). Maximum growth and consequently highest biosurfactant yield was observed in the sucrose supplemented growth medium followed by dextrose.

**Table 1:** Comparison of various physiochemical properties of biosurfactant produced by strain AR2 while growing on different carbon substrates

**Property**	**SMSM**	**DMSM**	**MMSM**	**LMSM**	**GMSM**	**SoMSM**
Initial surface tension (mN/m)	64.1 ± 1.6	64.5 ± 0.1	64.5 ± 1.6	64.1 ± 1.3	65.8 ± 1.3	64.2 ± 1.2
Final surface tension (mN/m)	30.0 ± 0.8	31.1 ± 0.2	35.7 ± 0.1	35.1 ± 0.5	35.3 ± 0.8	37.3 ± 0.4
CMC (mg/l)	80.0	80.0	100.0	100.0	100.0	110.0
Final Biomass (mg/l)	826.4 ± 116.6	770.4 ± 87.6	629.9 ± 64.9	571.7 ± 87.2	726.8 ± 88.2	430.2 ± 68.83
Biosurfactant yield (mg/l)	640.8 ± 54.2	539.9 ± 40.4	304.3 ± 24.4	264.7 ± 31.5	369.6 ± 20.1	172.7 ± 39.4
Productivity (mg/l/h)	10.6 ± 0.9	8.9 ± 0.6	5.1 ± 0.4	4.4 ± 0.5	6.6 ± 0.1	2.9 ± 0.7
% EI_24_ (Kerosene)	66.0	60.0	52.0	48.0	55.0	32.0

*SMSM*- sucrose minimal salt medium; *DMSM*- dextrose minimal salt medium; *MMSM*- maltose minimal salt medium; *LMSM*- lactose minimal salt medium; *GMSM*- glycerol minimal salt medium; *SoMSM*- sorbitol minimal salt medium.

### Characterization of biosurfactants

**Figure 2:**
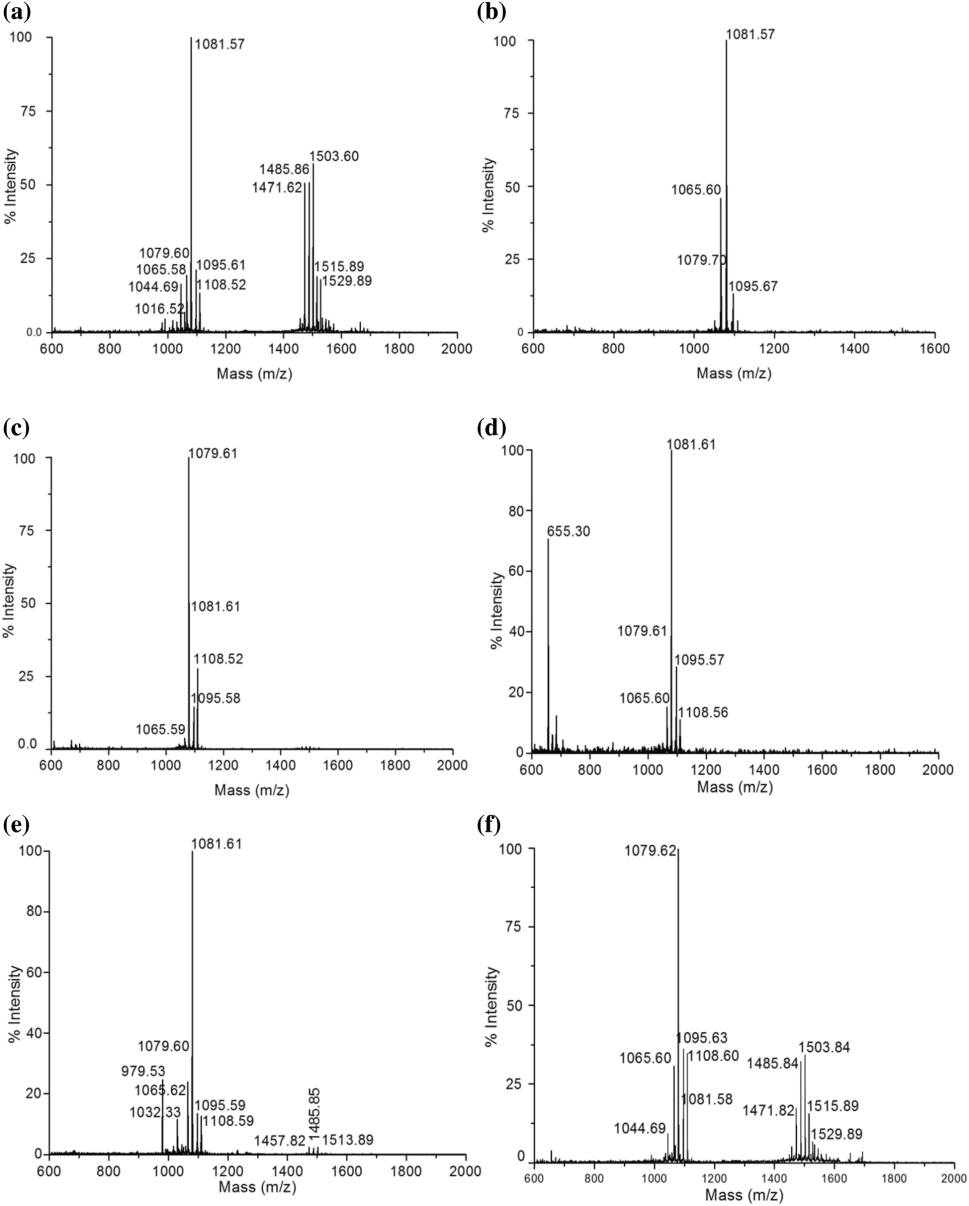
**MALDI-TOF mass spectra of purified lipopeptide produced by *B. amyloliquefaciens *** **strain AR2 while growing on (a) sucrose (b) sorbitol (c) lactose (d) maltose (e) glycerol and (f) dextrose.**

MALDI-TOF mass spectrometry was performed to illustrate the detail composition of purified biosurfactant produced by the strain AR2. The mass-spectra analysis of purified lipopeptides exhibited peaks which can be attributed to the sodium and potassium adducts. MALDI-TOF mass spectrometry indicated presence of two prominent and separate clusters of peaks when sucrose and dextrose was supplied as a carbon source. However, in the presence of other carbon source single cluster of peaks was observed (Figure 2). One cluster of peaks in the range of 1065.58 to 1108.52 m/z ratio corresponded to iturin isoforms and homologues. Another cluster of peaks was observed in the range of 1471.62 to 1529.89 m/z ratio corresponding to fengycin isoforms and homologues. In sucrose, dextrose and glycerol supplemented MSM small fraction of surfactin (1016.52, 1044.69 and 1032.33 m/z) was also observed along with iturin and fengycin. Strain AR2 produced C_15_ iturin as a most dominate lipopeptide when the bacterium was grown on sucrose, glycerol, maltose and sorbitol while C_14_ iturin was produced in the dextrose and lactose supplemented medium. Table 2 shows the mass numbers and assign details for the antifungal lipopeptide families observed in the MALDI-TOF mass spectra in the presence of different carbon source.

**Table 2:** **Assignment of all lipopeptides mass peaks obtained by MALDI-TOF mass spectrometry of biosurfactant produce by *B. amyloliquefaciens *** **strain AR2 while growing on different carbon substrate**

**Carbohydrate**	**Mass peak**	**Assignment**
Sucrose	1016.52, 1044.69	C_13_ surfactin [M + Na]^+^ Val-7, C_14_ surfactin [M + Na]^+^ Leu/lle-7
	1065.58, 1079.6, 1081.57, 1095.61, 1108.52	C_14_ iturin [M + Na]^+^, Asn-1; C_15_ iturin [M + Na]^+^, Asn-1; C_14_ iturin [M + K]^+^, Asn-1; C_15_ iturin [M + K]^+^, Asn-1; C_16_ iturin [M + K]^+^, Asn-1
	1471.62, 1485.86, 1503.60, 1515.89, 1529.89	C_15_ fengycin [M + Na]^+^, Ala-6; C_16_ fengycin [M + Na]^+^, Ala-6;C_17_ fengycin [M + H]^+^, Vla-6;C_17_ fengycin [M + K]^+^, Ala-6: C_16_ fengycin [M + K]^+^, Vla-6
Sorbitol	1065.60, 1079.70, 1081.57,1095.67	C_14_ iturin [M + Na]^+^, Asn-1; C_15_ iturin [M + Na]^+^, Asn-1; C_14_ iturin [M + K]^+^, Asn-1; C_15_ iturin [M + K]^+^, Asn-1
Lactose	1065.59, 1079.61, 1081.61, 1095.58, 1108.52	C_14_ iturin [M + Na]^+^, Asn- 1; C_15_ iturin [M + Na]^+^, Asn- 1; C_14_ iturin [M + K]^+^, Asn-1; C_15_ iturin [M + K]^+^, Asn-1; C_16_ iturin, [M + K]^+^, Asn-1
Maltose	1065.60, 1079.61, 1081.61, 1095.57, 1108.56	C_14_ iturin [M + Na]^+^, Asn-1; C_15_ iturin [M + Na]^+^, Asn-1; C_14_ iturin [M + K]^+^, Asn-1; C_15_ iturin [M + K]^+^, Asn-1; C_16_ iturin, [M + K]^+^, Asn-1
Glycerol	1032.33	C_13_ surfactin [M + K]^+^, Val-7
	1065.62, 1079.60, 1081.61, 1095.59, 1108.59	C_14_ iturin [M + Na]^+^, Asn-1; C_15_ iturin [M + Na]^+^, Asn-1; C_14_ iturin [M + K]^+^, Asn-1; C_15_ iturin [M + K]^+^, Asn-1; C_16_ iturin [M + K]^+^, Asn-1
	1471.82, 1485.84, 1513.84	C_15_ fengycin [M + Na]^+^, Ala-6; C_16_ fengycin [M + Na]^+^ Ala-6; C_16_ fengycin [M + Na]^+^, Vla-6
Dextrose	1044.69	C_14_ surfactin [M + Na]^+^, Leu/Ile-7
	1065.59, 1079.63, 1081.57, 1095.63, 1108.59	C_14_ iturin [M + Na]^+^, Asn-1; C_15_ iturin [M + Na]^+^, Asn-1; C_14_ iturin [M + K]^+^, Asn-1; C_15_ iturin [M + K]^+^, Asn-1; C_16_ iturin [M + K]^+^, Asn-1
	1471.82, 1485.84, 1503.84, 1515.89, 1529.89	C_15_ fengycin [M + Na]^+^, Ala-6; C_16_ fengycin [M + Na]^+^, Ala-6; C_17_ fengycin [M + H]^+^, Vla-6; C_17_ fengycin [M + K]^+^, Ala-6; C_16_ fengycin [M + K]^+^, Vla-6

Amino acid analysis of lipopeptide biosurfactant indicated presence of asparagines (Asn), glutamic acid (Glu), glutamine (Gln), alanine (Ala), proline (Pro), isoleucine (Ile), valine (Val), tyrosine (Tyr), threonine (Thr) , leucine (Leu), asparatic acid (Asp) and serine (Ser). Lipopeptides produced by strain AR2 while growing on sucrose, dextrose and glycerol as a carbon source have amino acid residues that are part of surfactin, iturin and fengycin. However, lipopeptide obtained from maltose, lactose and sorbitol based MSM showed presence of amino acid residues that can be assigned to iturin. Non-proteinous amino acid ornithine, an amino acid residue of fengycin, was also observed.

### Effect of different carbon source on antifungal activity of biosurfactant

Preliminary antifungal activity of biosurfactant was studied by well diffusion method. Concentrated cell free supernatant (CFS) obtained after 60 h of growth exhibited zone of inhibition against all the filamentous fungi tested in the present study. Zone of inhibition was observed to be influenced by the carbon source provided for the growth and biosurfactant production (Table 3). Maximum zone of inhibition was observed when sucrose or dextrose was provided as a carbon source. Minimum antifungal activity was observed when sorbitol was given as a carbon source. This observation may be attributed to relatively different biosurfactant concentration in CFS or different antifungal efficiency of lipopeptides. To ascertain the reason behind this observation, antifungal and fungicidal activity was studied by challenging fungi with equal concentration of purified biosurfactant.

**Table 3:** **Antifungal action of concentrated cell free supernatant collected from *B. amyloliquefaciens *** **strain AR2 while growing on different carbon substrate**

**Fungi**	**Diameter of inhibition zone (mm)**					
	**SMSM**	**DMSM**	**MMSM**	**LMSM**	**GMSM**	**SoMSM**
*F. solani* ATCC 36031	38.0 ± 2.0	37.0 ± 3.0	26.0 ± 4.0	30.0 ± 2.0	32.0 ± 4.0	18.0 ± 4.0
*F. oxysporum* MTCC 7229	40.0 ± 2.0	36.0 ± 5.0	25.0 ± 5.0	29.0 ± 4.0	27.0 ± 5.0	18.0 ± 5.0
*C. cladosporoides* ATCC 16022	34.0 ± 3.0	32.0 ± 3.0	19.0 ± 2.0	22.0 ± 4.0	20.0 ± 3.0	8.0 ± 5.0
*S. acremonium* ATCC 58636	43.0 ± 2.0	45.0 ± 2.0	33.0 ± 2.0	35.0 ± 5.0	36.0 ± 3.0	23.0 ± 3.0
*T. rubrum* MTCC 296	34.0 ± 4.0	30.0 ± 5.0	20.0 ± 5.0	24.0 ± 3.0	23.0 ± 1.0	9.0 ± 5.0
*M. gypseum* MTCC 4522	42.0 ± 5.0	40.0 ± 5.0	35.0 ± 3.0	36.0 ± 5.0	34.0 ± 6.0	25.0 ± 4.0
*A. alternata* MTCC 2724	36.0 ± 3.0	34.0 ± 5.0	24.0 ± 5.0	25.0 ± 3.0	24.0 ± 4.0	15.0 ± 5.0
*A. citri* MTCC 4875	36.0 ± 5.0	37.0 ± 3.0	23.0 ± 5.0	23.0 ± 5.0	25.0 ± 3.0	13.0 ± 4.0

*SMSM*-sucrose minimal salt medium; *DMSM*- dextrose minimal salt medium; *MMSM*- maltose minimal salt medium; *LMSM*-lactose minimal salt medium; *GMSM*- glycerol minimal salt medium; *SoMSM*- sorbitol minimal salt medium.

The purified antifungal lipopeptides produced by strain AR2 while growing on different carbon source exhibited different minimum inhibitory concentration (MIC) value (Table 4). Most efficient MIC was observed for the lipopeptides produced by strain AR2 while growing on sucrose and dextrose. The MIC of lipopeptides obtained from sucrose and dextrose as a carbon source was in the range of 125.0 –750.0 μg/ml, depending upon the targeted fungi. However, it was in the range of 250.0 –2000.0 μg/ml for the other carbon sources used in the present study. This suggests that the compositional variation in lipopeptides caused variation in the antifungal activity of biosurfactant.

**Table 4:** **Antifungal activity of purified lipopeptides produced by *B. amyloliquefaciens *** **strain AR2 while growing on different carbon substrate**

**Fungi**	**Minimum fungal growth inhibition concentration (μg/ml)**					
	**DMSM**	**MMSM**	**SMSM**	**LMSM**	**GMSM**	**SoMSM**
*F. solani* ATTC 36031	250.0	500.0	250.0	500.0	500.0	750.0
*F. oxysporum* MTCC 7229	250.0	250.0	500.0	500.0	500.0	750.0
*C. cladosporoides* ATCC 16022	750.0	750.0	1000.0	1000.0	1000.0	2000.0
*S. acremonium* ATCC 58636	125.0	125.0	250.0	250.0	250.0	500.0
*T. rubrum* MTCC 296	750.0	750.0	1000.0	1000.0	1000.0	2000.0
*M. gypseum* MTCC 4522	125.0	125.0	500.0	500.0	250.0	500.0
*A. alternata* MTCC 2724	500.0	500.0	750.0	750.0	750.0	2000.0
*A. citri* MTCC 4875	500.0	500.0	500.0	500.0	500.0	750.0

*SMSM*-sucrose minimal salt medium; *DMSM*-dextrose minimal salt medium; *MMSM*-lactose minimal salt medium; *LMSM*-lactose minimal salt medium; *GMSM*- glycerol minimal salt medium, *SoMSM*- sorbitol minimal salt medium.

**Figure 3:**
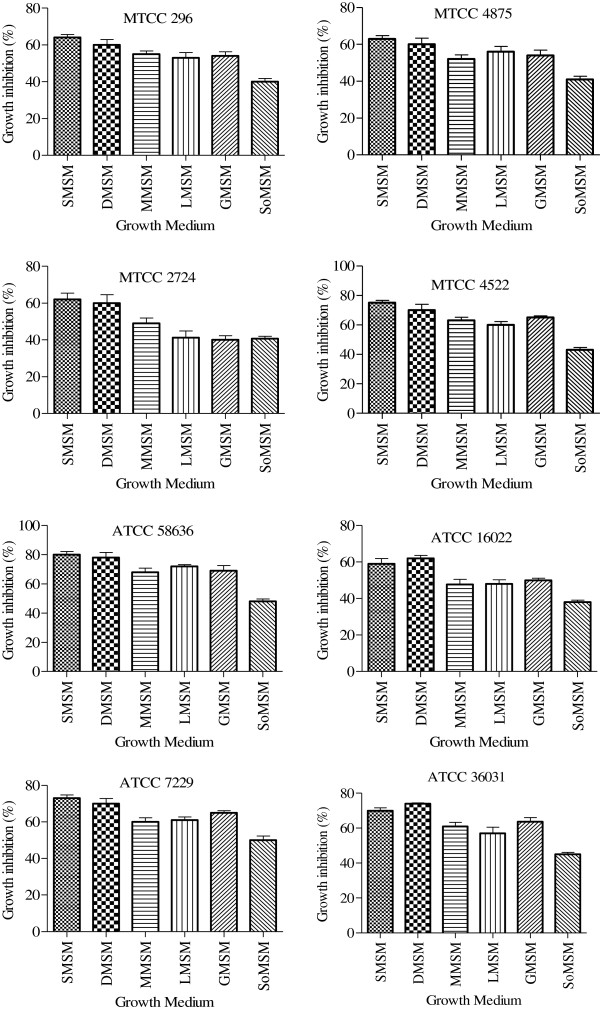
**Fungal mycelium growth inhibition by lipopeptides produced by *B. amyloliquefaciens *strain AR2 while growing on different carbon substrate.** Results are expressed as mean ± SD of three independent experiments performed in five replicates.

Lipopeptides showed mycelium growth inhibition activity at sub-MIC (i.e. ½ MIC). However, mycelium growth was totally absent at MIC of lipopeptides. Mycelium growth inhibition by biosurfactant was observed to be influenced by the carbon source provided for the growth. The most efficient growth inhibition was observed when sucrose and dextrose was supplied as a carbon source (Figure 3). Growth inhibition activity of lipopeptides produced on carbon sources namely sucrose, dextrose, maltose, glycerol and lactose was significantly (p < 0.05) superior than the lipopeptides produced on sorbitol supplemented MSM.

Challenging the fungal conidia with antifungal lipopeptides at the concentration two times the MIC decreased the colony forming unit significantly. Biosurfactant produced by strain AR2 while growing on sucrose decreased cfu/ml by more than 80% in 12 h of incubation. Interestingly, similar decrease in the viability of conidia was also observed, upon treatment with the biosurfactant obtained from different carbon source.

## Discussion

Lipopeptides biosurfactants from *Bacillus* species are renowned as one of the most efficient microbial surfactants. They are produced as a mixture of homologues and isoforms [5]. Variation present among cyclic lipopeptides makes it difficult to purify as well as to identify different families of lipopeptides and their homologues. The literature suggests that variation prevalent among lipopeptides biosurfactants is highly dependent on strain, culture condition and growth media composition [6-8]. Variation in the form of homologues and isoforms not only influence lipopeptides physiochemical properties but also its biological activities [6]. The carbon source provided for growth is utilized by Tricarboxylic acid (TCA) cycle. Intermediates from TCA cycle becomes part of amino acid and lipid moieties of lipopeptides, however little is known regarding the biosynthesis of the lipid moieties of the lipopeptide molecules [1].

Water soluble carbon sources used in the present study are commonly employed for biosurfactant production [6,12]. However, there is no detailed description of physiochemical and biological properties of lipopeptide produced in the presence of different carbon source. In the present study different carbon sources were supplied to the strain AR2, thus change in lipopeptide composition and yield was expected. The nature of carbon source directly influences the growth of biosurfactant producers. This in turn influences yield and the type of biosurfactant. Dextrose is the most favored carbon source for all living organism, while sucrose is one of the commonly used water soluble carbon source for biosurfactant production. In *B. amyloliquefaciens* dextrose supports a faster growth and biomass accumulation as compared to maltose [13]. A Simple sugar like dextrose is readily utilized by *Bacillus* cells for growth, thus leading to a rapid increase in the biomass. In the present study, also stain AR2 exhibited good growth and rapid biomass accumulation when dextrose was supplied as a carbon source. However, in the presence of maltose growth was comparatively retarded.

Liu et al. (2013) reported sucrose as the best carbon source among the water soluble carbon sources like glucose, galactose, maltose, glycerol, mannitol, soluble starch and dextrin for biosurfactant production [12]. Presence of array of glycoside hydrolases in *B. amyloliquefaciens* species bestows these microorganisms to utilize diverse range of carbohydrate [14]. Several studies have shown that in *Bacillus* species biosurfactant production is directly correlated to the biomass production [6,10,12]. Generally substrate supporting good growth also supports maximum biosurfactant production. In the present study, maximum biomass and biosurfactant yield was observed when sucrose was supplied as a carbon source. This observation is contrary to the report where glycerol was highlighted as the most efficient carbon source for biosurfactant production [6].

Genome analysis of *Bacillus amyloliquefaciens* GA1 exhibited presence of gene clusters directly involved in the synthesis of the cyclic lipopeptides surfactin, iturin and fengycin [15]. Arrebola et al. observed that *B. amyloliquefaciens* PPCB004 can produce surfactin, iturin and fengycin while growing on sucrose based growth medium [16]. However, *B. amyloliquefaciens* strain B94 was only able to produce iturin when grown in Luria-Bertani (LB) broth [17]. Another stain, *B. amyloliquefaciens* LSCo4 produced fengycin while growing on LB supplemented with 2% crude oil [18]. In the present study, surfactin, iturin and fengycin were produced by strain AR2 when sucrose, dextrose or glycerol was supplied as a carbon source. The observed variation between the previous reports and the present study can be correlated with the different growth media composition or strain.

Variation in the carbon source changed the type of lipopeptides produced by strain AR2. Interestingly, amino acid moieties of lipopeptides (surfactin, iturin and fengycin) remained the same for all the carbon sources (except sucrose, dextrose and glycerol for which changes in the amino acid moieties at position 7 were observed), though variation in the fatty acid chain length was observed. Lipopeptides biosynthesis is a complicated process involving significant role of two component systems and quorum sensing. In *Bacillus*, lipopeptide biosurfactants are synthesized by the non-ribosomal peptide synthatase (NRPS) pathway that utilizes the multiple-carrier thiotemplate mechanism [1,5]. Valteer et al. reported that in *B. subtilis* C-1 the production of surfactin, iturin and fengycin depends on the growth period under similar growth media and conditions [8]. The expression of the surfactin genes is associated with increased cell densities and occurs especially in the transition from exponential to stationary growth phase, whereas the biosynthesis of fengycin and iturin usually occurs later in the stationary phase [8]. As compared to surfactin regulatory network, little information is available about the regulation of iturin and fengycin biosynthesis [8]. In the present study, the sole production of iturin under the influence of sorbitol, lactose and maltose as a growth substrate suggests that along with influence of the growth phase, some unknown mechanism may also be involved.

The surface activity of lipopeptides biosurfactants depends on their structural components, namely their hydrophilic and hydrophobic groups and their spatial orientation. Surface reducing activity of a biosurfactant defines its efficiency. According to Mulligan, an efficient biosurfactant can reduce the surface tension of water to 35mN/m [19]. In this respect the biosurfactant produced by strain AR2 constitutes efficient microbial surfactant. Biosurfactant from strain AR2 was able to reduce the surface tension of water in the range of 30 - 37 mN/m depending upon its composition. Das et al. observed that different lipopeptides have different surface tension reducing ability [6]. The surface activity of lipopeptides like surfactin is influenced by the amino acid residues and fatty acid chain [20]. In surfactin substituting the hydrophobic amino acids like L-valine by more hydrophobic residues like leucine or iso-leucine increased the surface tension reducing activity and decreased the CMC [21]. Yakimov et al. reported that branching of fatty acid chain decreases surface tension reducing ability of lipopeptides [22]. As compared to iturin, fengycin has longer fatty acid chain and more hydrophobic amino acid residue. Thus, lipopeptide with iturin and fengycin displayed better surface activity as compared to the biosurfactant consisting of iturin only.

Lipopeptides obtained from sucrose and dextrose exhibited higher emulsification index as compared to the other carbon sources. This observation may be attributed to higher biosurfactant concentration in sucrose and dextrose supplemented MSM. Emulsification index has been reported to be proportional to the surfactant concentration till CMC value is attained [23]. Contrary to the present observation, Das et al. reported that the lipopeptide biosurfactants produced by *Bacillus sp.* grown on glycerol based MSM has higher emulsification power than the ones obtained from dextrose and sucrose [6].

Lipopeptides are known as effective antimicrobial against bacteria, fungi and viruses [5]. In the present study, CFS with lipopeptides was used for well diffusion assay after concentrating it with 3kD cut off membrane, so that the concentration of antifungal biosurfactant increases in the broth. Concentrating CFS helps to screen out even those microorganisms that are producing small amounts of antifungal compounds in the provided growth medium. Lipopeptides are low molecular weight microbial surfactants [24]. However, at concentrations above the CMC, the low molecular weight biosurfactants associate to form supramolecular structures. This characteristic of the biosurfactant has been used to concentrate and recover lipopeptides biosurfactants from complex fermentation medium [25].

MIC value of lipopeptides biosurfactant against a particular fungus was observed to be different. This observation may be attributed to different composition of lipopeptides produced by strain AR2 while growing on different carbon substrates. Most efficient antifungal activity was observed when sucrose or dextrose was provided as a carbon source. The strain AR2 produced mixture of iturin and fengycin, when sucrose or dextrose was provided as a carbon source. Iturin and fengycin are known to have strong antifungal activity against filamentous fungus when they act in a synergistic manner [26]. Iturin has strong antifungal activity against a wide variety of yeast and fungi. Antifungal activity of iturin relies on the membrane permeabilization properties [27]. Fengycin have a strong fungitoxic activity against filamentous fungi. The action of fengycin is less well known as compared with other lipopeptides biosurfactant [28]. They readily interact with lipid layers and to some extent have the potential to alter cell membrane structure and permeability in a dose-dependent way.

Antifungal activity of lipopeptides against dermatophytes like *Trichophyton rubrum* can be exploited in topical formulations effective against skin diseases. Antifungal activity of strain AR2 against phyto-pathogen like *F. oxysporum, F. solani, A. citri* and *A. alternate* can be exploited for developing bacterial bio-control agent. Apart from antifungal activity, strain AR2 also demonstrated few plant growth promoting traits namely phosphate solubilization, catalase, siderophores and auxin production (data not shown). Spore forming ability, ubiquitous and non-pathogenic nature, along with antimicrobial activity makes several species of genus *Bacillus* as a potential bio-control agent [28]. The ability of the strain AR2 to act as a bio-control agent will depend on the carbon source available in the root exudes. This implies that rhizosphere conditions can drive the physiological activity of strain AR2 to a particular state that in turn could favor the production of one or more specific lipopeptide biosurfactants.

## Conclusions

Lipopeptides biosurfactants production in *B. amyloliquefaciens* strain AR2 is significantly influenced by carbon substrate provide for the growth. Different water soluble carbon source not only influenced the biosurfactant yield but also exerted note worthy effect on lipopeptides composition, surface activity, emulsification behavior and antifungal activity. Among the water soluble carbon sources namely dextrose, sucrose, glycerol, maltose, lactose, and sorbitol, sucrose was the most suitable carbon substrate for biosurfactant production. Maximum biosurfactant yield and antifungal activity was observed when sucrose was given as a carbon source to the strain AR2. Thus, the carbon source is important not only for determining the yield but also the type of biosurfactant. Antifungal activity of lipopeptides produced by strain AR2 can be utilized for developing environment friendly fungicides. Strain AR2 can also be exploited as a bacterial bio-control agent against fungal phyto-pathogens.

## Materials

### Filamentous fungal species and culture conditions

The filamentous fungal strains selected for the study are known to cause economic loss by infecting human, animals and plant, food contamination, corrosion of metals and many other nuisances for mankind [29]. Fungi were obtained from the Microbial Type Culture Collection and Gene Bank (MTCC) IMTECH, India and American Type Culture Collection (ATCC), USA. All the strains (namely *Fusarium solani* ATCC 36031, *F. oxysporum* MTCC 7229*, Cladosporium cladosporoides* ATCC 16022, *Scopulariopsis acremonium* ATCC 58636*, Microsporum gypseum* MTCC 4522, *Alternaria alternata* MTCC 2724, *Alternaria citri* MTCC 4875 and *Trichophyton rubrum* MTCC 296) were grown and preserved as recommended by MTCC and ATCC.

For conidia formation the fungi were grown on Potato dextrose agar (PDA) at 30°C for 7–10 days. Conidia were harvested by flooding the surface of the agar plates with 5 ml PBS (pH 7.2) containing 0.025% (v/v) Tween 20 and shaking gently. The conidial suspension was recovered and dispensed into a 10 ml sterile glass vials. The conidia were counted using a haemocytometer and adjusted to the required concentration in RPMI 1640 (with L-glutamine; without sodium bicarbonate) buffered to pH 7.0 with 0.165 M 4-Morpholinepropanesulfonic acid sodium salt (MOPS).

### Isolation of biosurfactant producing bacterium

The biosurfactant producing bacterium used in this study was isolated from industrial effluent collected from Buddha Nullah, Ludhiana, India using serial dilution plating method on Nutrient agar (NA) plates. The strain AR2 was selected as it was able to reduce the surface tension of Nutrient broth (NB) to 30 mN/m within 24 h of growth. The bacterium was identified as *Bacillus amyloliquefaciens* (Genbank databank Accession no. KF289765) by morphological, biochemical and physiological studies (API 20NE Kit, Bio Merieux, Mercy, France). Its identity was further confirmed by 16S rRNA gene sequencing. NA and NB were used for culture maintenance and preparation of inoculums, respectively.

### Biosurfactant, production, purification and characterization

Biosurfactant production was carried out in 2 l Erlenmeyer flasks containing 500 ml aliquots of minimal salt media (MSM). MSM was composed of (per l): 4 g NH_4_NO_3_, 4 g KH_2_PO_4_, 5.68 g Na_2_HPO_4_, 0.78 mg CaCl_2_, 197.18 mg MgSO_4_.6H_2_O, 1.112 mg FeSO_4_ and 0.1 ml of trace element solution (TES). TES composition (per l): 2.32 g ZnSO_4_ 7H_2_O, 1.78 g MnSO_4_ 4H_2_O, 0.56 g H_3_BO_3_, 0.78 CuSO_4_ 5H_2_O, 0.39 g Na_2_MoO_4_ 2H_2_O, 0.42 g CoCl_2_ 6H_2_O 0.10 g EDTA, 0.004 g NiCl_2_ · 6H_2_O and 0.66 g KI. Carbon source namely sucrose, dextrose, maltose, lactose, glycerol and sorbitol was added in such a manner that C: N ratio remains constant at 15.55. The pH of media was adjusted to 7.0 ± 0.5 with 1 N HCl and 1 N NaOH. The flasks were inoculated with overnight grown seed culture at 2% (v/v) and incubated at 30˚C with the agitation rate of 200 rpm. Growth was monitored by measuring absorbance at 600 nm and dry biomass weight method.

After cultivation, the cells were harvested by centrifugation at 8000 rpm for 10 min. The CFS was acid precipitated with 6 N HCl to pH 2 and incubated overnight at 4˚C. The biosurfactant was extracted from precipitate with methanol and then concentrated with the help of a rotary evaporator. The crude antifungal compound was further purified by gel filtration on Sephadex LH-20 column (60 cm x 1 cm) with methanol as elutent. Methanol was evaporated under reduced pressure and active fraction (determined by performing zone of inhibition assay) was lyophilized. Lyophilized active fraction was weighed to determine the yield of antifungal biosurfactant.

The active fraction was characterized by MALDI-TOF mass spectrometry [30,31]. MALDI-TOF mass spectra were recorded by using Applied Biosystems Voyager MALDI-TOF instrument containing a 337-nm nitrogen laser for desorption and ionisation. Lyophilized fraction was dissolved in methanol (~1 mg/ml). An aliquot of ~2 μl was mixed with an equal volume of matrix medium (solution of α-cyano-4-hydroxycinnamic acid in 70% aqueous acetonitrile containing 0.1% v/v TFA). The well mixed sample was spotted on a target plate, dried and placed inside the sample cabinet of the instrument. 20 kV of acceleration voltage was applied to accelerate the molecules. Positive-ion detection and the reflector mode were used. The molecular mass gate of 100 Dalton was provided to avoid noise for the better precision.

Amino acid analysis was performed according to Waters Pico Tag method by pre-column derivatization with Phenylisothiocyanate. The surface tension was measured at 25°C using a duNouy tensiometer (CSC Scientific Company Inc., USA) based on ring detachment method [31]. Emulsification activity of lipopeptide against kerosene was measured following the method given by Das et al., [6]. Briefly, equal volumes of CFS and kerosene was mixed, and vortexed at high speed for 5 min followed by incubation at 25°C for 24 h. The emulsification index value (EI_24_) was then calculated using the formula: EI_24_ = (Height of emulsion layer/Height of the total mixture) × 100.

### Antifungal activity of biosurfactant

The preliminary antifungal activity of biosurfactant was examined following method given by Rautela et al. [32]. In brief, the CFS obtained after 60 h of growth was concentrated to 50% (v/v) by 3kd MWCO Amicon Ultra-4 centrifugal filters (Millipore Corporation, USA). Concentrated CFS was used for performing well diffusion assay against all the fungi used in the present study. Fungi were incubated at 37°C for 5 d and presence or absence of zone of inhibition was observed.

Antimicrobial activity of any compound is best appreciated by its MIC. MIC of biosurfactant was determined by 2, 3-bis (2-methoxy-4-nitro-5-(sulfophenyl)-5-[(phenylamino) carbonyl]-*2H*-tetrazolium- hydroxide (XTT) reduction test [33]. Wells of 96-well flat bottom microtiter plates were filled with 100 μl of RPMI 1640 medium containing lipopeptide at different concentrations. Further each well was inoculated with 100 μl of conidia suspension so that the final concentration of inoculums is 1 × 10^4^ cfu/ml. The plates were incubated for 36 h at 37°C. Then, 50 μl of XTT-menadione (0.5 g/L XTT, 1 μM menadione) was added to each well and further incubated for 2 h at 37°C. After incubation 100 μl of the colored supernatant was transferred into the wells of a new microtiter plate. Absorbance of each well at 492 nm was measured by microtiter plate reader (Senergy 2 Multi-mode microplate reader, BioTek, USA). Un-inoculated wells containing RPMI 1640 with and without lipopeptide was used as controls. Reading of negative control was subtracted to obtain real values. MIC was described as a lowest concentration at which no change in colour was observed while percentage growth inhibition at different biosurfactant concentrations for each fungus was calculated as: Growth inhibition (%) = (A_0_−A_C_) × 100 /A_0_ where A_C_ represents the absorbance of the well with lipopeptide biosurfactant and A_0_ the absorbance of the control well (without biosurfactant).

### Mycelium growth inhibition by biosurfactant

Mycelium growth inhibition was examined by placing 6 mm mycelium agar disc (taken from 5 days old plates) at the center of PDA plates containing antifungal lipopeptides at MIC and 1/2 MIC. All plates were incubated at 37°C for 5 days. Fungal growth was observed as an increase in colony diameter. Five replicate plates were used per assay. Percentage mycelium inhibition by lipopeptide was calculated by the formula: Mycelium growth inhibition (%) = (D_0_−D_C_) × 100/D_0_ where D_C_ represents the diameter of colony growing on lipopeptide amended plates and D_0_ the diameter of colony growing on control plates (without lipopeptides).

### Fungicidal activity of lipopeptides

Fungicidal activity of lipopeptide was estimated by colony forming units per ml (cfu/ml) count. Fungal conidia suspension (6 × 10^6^ cells/ml) in the presence (2 × MIC) and absence of cyclic lipopeptide was incubated at 37°C with an agitation rate of 200 rpm. The cfu/ml was determined by plating it on PDA at different time intervals.

### Statistical analysis

All experiments were performed in at least five biological replicates and the data were expressed as mean ± standard deviation. To define statistically significant, the data were analyzed using one- way ANOVA and the data were considered statistical significance if p < 0.05.

## Competing interests

The authors declare that they have no competing interests.

## Author’s contribution

AKS planned and performed the experiments. RR helped AKS in experiments. SSC supervised the work. AKS and SSC wrote the final version of the manuscript. The authors approved the final version of the manuscript.
